# Relationship between food quality and body size of common vole in different habitats

**DOI:** 10.1002/ps.70628

**Published:** 2026-02-06

**Authors:** Eva Jánová, Ladislav Čepelka, Josef Suchomel, Marta Heroldová

**Affiliations:** ^1^ Department of Forest Ecology Mendel University in Brno Brno Czech Republic; ^2^ Institute of Vertebrate Biology, Czech Academy of Sciences Brno Czech Republic; ^3^ Department of Zoology, Fisheries, Hydrobiology and Apiculture Mendel University in Brno Brno Czech Republic

**Keywords:** body size, habitat, *Microtus arvalis*, nitrogen content

## Abstract

**BACKGROUND:**

There is a close relationship between habitat, food and demographic parameters of common vole populations. The current study investigates the relationship between the quality of food consumed by adult voles in different habitats and vole body size. Populations of common voles were monitored within a small region under the same weather conditions in both forest and agricultural habitats. The quality of the food consumed was determined using near‐infrared spectroscopy. The indicator of food quality was the content of nitrogenous compounds. Other key variables were also considered: sex, reproduction, year and date of capture, and relative vole abundance in each plot.

**RESULTS:**

Food quality and body size were dependent on date and habitat. Voles from arable habitats, such as cereals, rape and alfalfa, were larger than those from less managed habitats (set‐aside, clearings and forests). Differences in body size did not appear to be directly related to food quality; voles in cereals had lower food quality than voles in clearings, rape, alfalfa and set‐aside. Individuals captured in 2008 were smaller than in other years, but the year of capture did not affect food quality.

**CONCLUSION:**

Diet quality is a limiting factor for body growth, but body size is influenced by factors other than food nitrogen content alone. © 2026 The Author(s). *Pest Management Science* published by John Wiley & Sons Ltd on behalf of Society of Chemical Industry.

## Graphical abstract

The body size of the common vole in crop fields (alfalfa, rape, cereals) is larger than in less‐cultivated habitats (forests, clearings, set‐aside) and is not related to food quality.

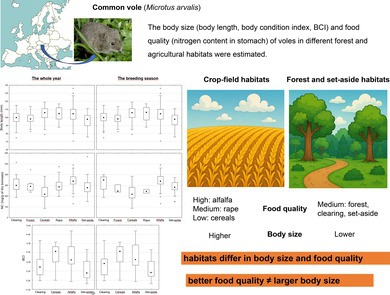

## 1 INTRODUCTION

The provision of adequate food and living space, both in terms of quality and quantity, is one of the fundamental needs of all animals. Small rodents exhibit relatively low mobility, and their home ranges are relatively small compared with those of larger mammalian species. Consequently, their relationship with the microhabitat is particularly close. The species composition and population dynamics of small mammal communities are influenced by local vegetation cover and food supply.^1^, ^2^ Voles are less mobile than other rodent species and are dependent on resources within a radius of a few tens of metres.^3^, ^4^, ^5^ Consequently, the composition of the voles’ diet closely reflects the species composition and phenological state of the local vegetation.

The common vole (*Microtus arvalis*, Pallas 1778) is the most abundant rodent species in agroecosystems across Central Europe.^6^, ^7^ This species prefers open steppe habitats and is a typical herbivore.^5^ Common vole populations are subject to interannual fluctuations in abundance. This is most pronounced in crops with high densities (e.g. alfalfa). However, like other small mammals, voles are found not only in their typical habitats, but also in environments that are not entirely natural for them, such as clearings and young forests.^8^

Environmental factors have been shown to influence morphological (body size) and physiological parameters such as reproduction, litter size, survival or lifespan of small rodents.^9^, ^10^, ^11^ Body size in small rodents is known to be influenced by reproduction, the phase of the population cycle and seasonal cohort membership.^12^, ^13^, ^14^ In small mammals, changes in body size have been described as a function of altitude,^15^, ^16^ precipitation^17^, ^18^ and food availability.^19^, ^20^

A limited number of studies from Lithuania have suggested that the body size or body condition index (BCI) of common vole may vary according to habitat within the same region.^21^, ^22^ In general, voles in more disturbed habitats in Germany^23^ have a higher average body weight and undergo greater seasonal fluctuations than those in less disturbed habitats. These size differences may be caused by variations in abundance and the associated Chitty effect, as observed in Spain.^24^ There is a lack of studies that have compared body size in Central Europe at the local level, in populations living side by side, i.e. under the same climatic conditions, but in different types of habitats. Furthermore, little research has been conducted on how food quality affects body size in small mammals living in different habitats within the same geographical area, and whether any observed changes in body size can be attributed to variations in food quality.

Currently, near‐infrared reflectance spectroscopy (NIRS) is one of the methods used to determine the quality of food consumed by small mammals in forest ecosystems^25^ and agricultural habitats.^26^ In this study, the NIRS approach is used to analyse the food quality of common voles living in different habitats.

The aim of this study was to determine the differences in food quality and body size of vole populations living in neighbouring but different habitats (both agricultural and forested). On the one hand, we assume that the quality of food varies across habitats, which can be reflected in differences in body length. On the other hand, the common vole, an original steppe species, is well adapted to steppe‐like conditions in habitats such as set‐aside land and alfalfa fields. In these habitats, voles should be larger than in less typical habitats (e.g. rape fields or forests), regardless of food quality. However, variation in the body length of the common vole is a more complex issue. In addition to environmental conditions, we should keep in mind that body length, like other demographic parameters, can also be entirely and strictly influenced by seasonality and year‐to‐year fluctuations. There, we assume that the Chitty effect^12^ and the trade‐off between reproduction and growth^13^ can influence body length through its connection with density cycles and population abundance.

Based on the above, we tested the following hypotheses:

- Food quality and body length in the vole population vary depending on the habitats.
- Voles in habitats with higher‐quality food are larger.
- Body length and food quality vary between years, seasons, and common vole abundance.

## 2 MATERIAL AND METHODS

### 2.1 Study area and sampling methods

Small mammals were monitored in the flat agricultural region of southern Moravia (Czech Republic), in the northern part of the Pannonian basin, at elevations of 180–250 m. The local landscape has been shaped by intensive agricultural production for centuries, resulting in a mosaic dominated by arable land with smaller areas of forests and meadows.

Forest and agricultural habitats were monitored in two independent projects to determine the impact of small mammals on individual agricultural crops and forest stands. We have previously analysed the population dynamics of rodent communities in agricultural and forest habitats.^25^, ^27^, ^28^, ^29^ In this study, we examine the relationship between body length and food quality in the common vole in its typical and less common habitats. Monitoring of small mammals in forest habitats was carried out between 2004 and 2010 in the three large, isolated forest complexes (>60 ha): Lednice (48.8083 N, 16.7848 E), Vranovice (48.9567 N, 16.5937 E) and Židlochovice (49.0401 N, 16.7126 E). In all cases, these are mixed forests with a predominance of oak.^25^, ^30^ Trapping took place usually five times a year, always after about 2–2.5 months, both in forest stands (habitat forest) and in clearings (habitat clearing).^25^

The agricultural habitats (6–13 plots for each crop and year) were in the area between Židlochovice and Břeclav (GPS coordinates 48.7945–49.0531 N, 16.5736–16.6179 E). The first part of the trapping in agroecosystems took place in 2004–2006, in the habitats of winter wheat, spring barley, winter rape and perennial alfalfa.^27^ In 2008–2010, set‐aside was added to the already monitored habitats.^29^ Small mammals were captured here every 5–7 weeks. Permanent stands of alfalfa and set‐aside, as well as winter wheat and winter rape, were monitored year‐round. Although winter wheat and rape are not officially considered permanent crops, they are a source of food for small mammals even in the post‐harvest period, when the remnants of their vegetation remain in the field. Moreover, in central European conditions, these crops are sown very soon after harvest. Barley was monitored from the grown to harvest, usually from April to July.

Sampling was performed by snap‐trapping because the secondary purpose of the trapping was to obtain material for screening of rodent‐borne zoonoses, such as *Mycobacterium avium*, hantavirus, arena virus and cowpox viruses.^31^, ^32^, ^33^, ^34^, ^35^, ^36^ The snap‐trapping method allowed us to study the food composition in the stomachs of small mammals. Snap‐traps were baited with fried wicks (soaked in fat and flour) smeared with peanut butter. In each habitat, 25 or 50 snap‐traps were laid about 3 m apart. Traps were checked daily and left in place for three nights. For common voles and each habitat and trapping event, the relative abundance (rA) was determined as the number of individuals caught in a particular habitat per 100 trap‐nights.

All captured individuals were identified to species level, weighed, sexed, measured and dissected. If the stomach was of sufficient size, it was removed and dried for subsequent analysis of food quality. Females with embryos or visible maculae cyaneae were considered reproductively active, whereas those without these signs of reproduction were recorded as inactive. Males with testes 10 mm or more in length were considered reproductively active, whereas those with smaller testes were considered inactive.^37^

The work complied with Council Directive 86/609/EEC regulations on the experimental use of animals and was carried out in accordance with all applicable ethical rules.

### 2.2 Analysis of the nitrogen content in the stomach

NIRS was used to determine the nitrogen content (NC) of the stomach. NC was estimated as mg per g of dried stomach content. A previously developed methodology and calibration model, validated and used for the determination of NC in rodent stomachs was applied.^38^, ^39^, ^40^ Briefly, stomachs were dried, ground with sandpaper and analysed using a near‐infrared spectrophotometer FOSS NIR System 6500 (Foss Nirsystems, Laurel, MD, USA) in the 1100–2500 nm wavelength range. The obtained spectra were analysed using the calibration model.^38^

We suppose that the quality of the analysed food consumed just prior to capture is not directly related to the body size of the individual. However, if some relationship does occur, it should be tested (see below). Nevertheless, the observed NC values can well describe the nutritional parameters of a given habitat in each period. In general, these NC values are representative of the nutritional parameters of the habitat because of the large number of individuals captured at the sites. We suppose that NIRS results are only minimally affected by baiting. Consumption of bait may occur to a limited extent, but the trap mechanism is usually so sensitive that the individual is killed on the first attempt to taste or pull the bait. In addition, the same bait was used everywhere, and the probability of its consumption is the same in all habitats and years.

### 2.3 Statistical analysis

The relationship between food quality, body size and other individual, population and environmental parameters was analysed. Because the different proportions and size/age of juveniles in the habitats could influence the results, only reproductively active individuals were analysed. Body length was chosen as the parameter describing the body size of adult individuals.

For males, BCI was also determined as an indirect indicator of body condition as body weight divided by body length. The BCI was not estimated for females because their body weight is affected by pregnancy or energy‐intensive lactation. However, BCI can be strongly influenced by the randomness of the filling of the digestive tract at the time of capture (it can be up 23% of body weight).^41^ It can be assumed that body length is more likely to reflect conditions during the growth period of the organism, whereas BCI may be a much better indicator of the current habitat characteristics and the condition of an individual animal. The power and significance of the BCI analysis is also affected by the much smaller sample size and the fact that BCI describes only adult males, not females, which may have a different body size pattern.

For statistical analysis, the voles captured in winter wheat and spring barley were combined into the more numerous ‘Cereals’ category. The phenology, characteristics and chemical composition of these crops are very similar; spring barley grows very quickly after sowing and soon approaches that of wheat in terms of quantity and type of biomass, after which both cereals have a similar phenology. Cereals have similar agronomic management (type, method and timing of interventions, including fertiliser and pesticide application) and similar weeds.^42^ The newly created category ‘Cereals’ can be considered as consistent and at the same time the results are sufficiently robust.

Analysis of NC and body size was carried out only for years in which both forest and field habitats were sampled, and only for habitats where at least eight individuals could be used in the analyses. Common vole abundance is subject to natural variation. The years 2006 and 2009 were characterised by low densities and the low number of individuals captured did not allow these years to be included in the analysis. Consequently, only the years 2004, 2005, 2008 and 2010 could be analysed (the number of individuals analysed is summarised in Table 1). Because of the limited number of adult males in both rape and forest, it is not possible to analyse BCI in these habitats.

**Table 1:** Number of adult common voles used for analyses of body length, body condition index (BCI; adult males only) and stomach nitrogen content in each habitat in the whole year (WY) and during the breeding season (BS)

	Body length			BCI			Nitrogen content			Max. rA
	WY	BS	Mean	WY	BS	Mean	WY	BS	Mean	
Numbers and values for each analysed year separately										
2004										
Alfalfa	38	38	106.7	0	0	NA	38	38	337.9	35.7
Cereals	16	16	102.7	3	3	0.302	16	16	229.5	6.2
Clearing	15	12	101.0	6	6	0.268	6	5	306.9	2.2
Forest	4	4	94.3	NA	NA	0.252	1	1	263.2	1.7
Rape	4	4	108.0	NA	NA	NA	4	4	267.2	4.1
Total	77	74	104.2	10	10	0.277	65	64	302.9	
2005										
Alfalfa	42	42	104.3	5	5	0.331	42	42	357.7	37.1
Cereals	27	27	106.4	4	4	0.269	27	27	233.4	10.5
Clearing	17	12	96.6	8	4	0.251	12	7	324.3	2.2
Forest	7	7	101.1	NA	NA	0.287	7	7	305.6	1.7
Rape	3	3	110.7	NA	NA	NA	3	3	288.9	3.1
Total	96	91	103.4	21	17	0.281	91	86	310.1	
2008										
Alfalfa	14	14	100.1	6	6	0.278	14	14	305.7	7.0
Cereals	5	5	99.6	3	3	0.347	5	5	238.2	2.0
Clearing	17	17	95.7	6	6	0.225	0	0	NA	1.7
Rape	3	2	96.7	NA	NA	NA	3	2	273.0	1.0
Set‐aside	19	17	93.3	5	4	0.232	19	17	280.3	6.0
Total	58	55	96.3	21	19	0.258	41	38	283.3	
2010										
Alfalfa	6	4	99.7	2	1	0.281	6	4	359.6	4.5
Cereals	9	9	104.6	6	6	0.319	9	9	231.6	2.0
Clearing	34	33	102.8	11	10	0.292	1	1	193.8	1.7
Forest	3	3	106.7	NA	NA	0.232	0	0	NA	1.7
Rape	3	3	102.0	NA	NA	NA	3	3	374.7	1.5
Set‐aside	16	13	100.1	8	8	0.253	17	14	318.8	4.5
Total	72	65	102.0	29	27	0.284	36	31	304.9	
All years	302	285	101.9	81	73	0.276	233	219	302.6	
Numbers and values for all analysed years together										
Alfalfa	100	98	104.3	13	12	0.299	100	98	343.0	35.7
Cereals	57	57	104.5	16	16	0.309	57	57	232.5	10.5
Clearing	83	74	99.7	31	26	0.264	19	13	311.9	2.2
Forest	14	14	98.6	NA	NA	NA	8	8	300.3	1.7
Rape	13	12	109.6	NA	NA	NA	13	12	298.3	4.1
Set‐aside	35	30	96.5	13	12	0.246	36	31	298.5	6.0

The mean values of body length, BCI and stomach nitrogen content and maximum values of relative abundance (Max rA) are provided for each habitat and year. BCI in rape and forest was not analysed (NA) because of the small sample size.

Statistical analysis was performed in a series of steps on sets of different sizes. First, all captured adults were analysed. This analysis included data from the whole year, but only perennial habitats were monitored during the winter. Therefore, the analysis may be affected by variation in overwintering conditions and winter food sources within some habitats. For this reason, in a second step, the analysis was restricted to the breeding season, which overlaps with the vegetation season in Central Europe. The breeding season was defined as the period from 10 April to the end of September, when most adults are reproductively active. The other reason for this seemingly strict temporal limitation of the breeding season is that previous monitoring was usually carried out in February and the subsequent monitoring in November. During these months, reproduction was rarely detected, and vegetation was in the resting phase. However, it should be noted that only 17 individuals were captured during the non‐breeding season, which is fewer than 6% of the individuals captured during the breeding season (Table 1). In both study data sets, the number of individuals used for body size analysis exceeds the number used for food quality analysis. This discrepancy can be explained by the fact that some individuals’ stomachs could not be used for NIRS analysis (stomachs were too empty).

The variables used for statistical evaluation were the following categorical variables: year, habitat, sex (sex was not analysed in the BCI analyses where only males were included) and breeding or non‐breeding season (this variable was only included in the full year analysis). The continuous variables used were: date (the capture day's number since the beginning of the year) and rA per monitored plot (number of all captured individuals, including juveniles, in the specific plot per 100 trap‐nights). In the analysis of the variables influencing body length, in addition to the other variables, the continuous variable NC was also used.

The effect of the previous variables on body length, BCI and NC was then analysed by General Linear Modelling (GLM, normal distribution with log link function). In cases where the effect of habitat or year was detected, specific differences were analysed using the post‐hoc Tukey HSD test. We recognise that the use of post‐hoc tests designed for one‐way analysis of variance may be somewhat misleading and simplistic, because other factors are neglected here. All statistical analyses were performed using Statistica, v.14.^43^

## 3 RESULTS

**Figure 1:**
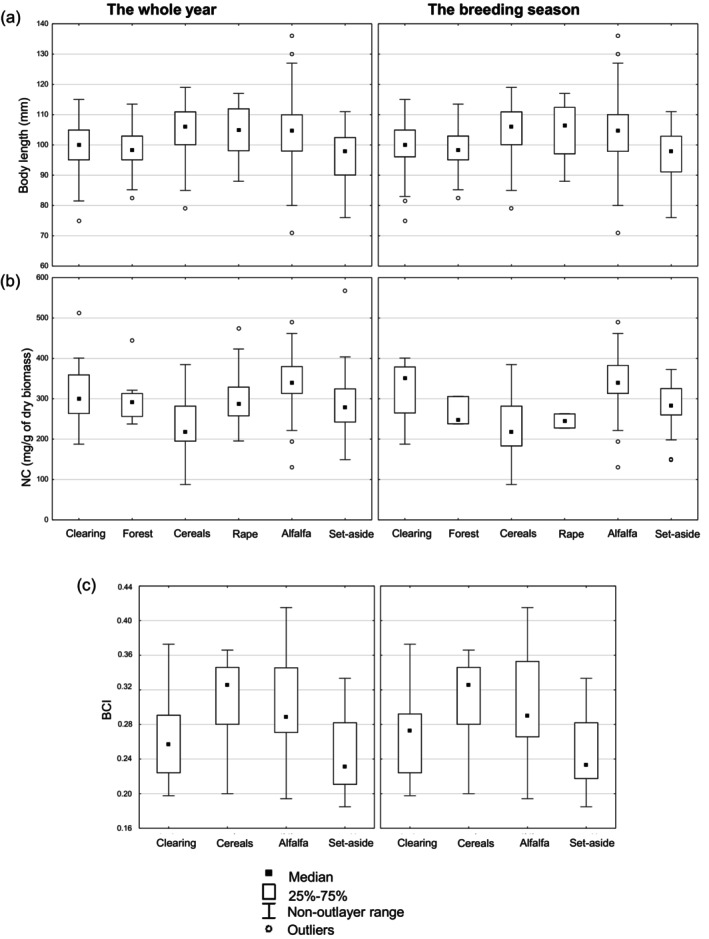
(a) Body length (mm), (b) stomach nitrogen content (NC as mg/g of dry biomass) and (c) body condition index (BCI) values for common vole in the analysed habitats.

The values of the proportion of variance explained by the GLM model (*R* ^2^) and regression coefficients of quantitative predictors are summarised in Table 2. The effect of habitat on NC values, body length and BCI was found to be significant both for the whole year and for the breeding season only (Table 3). NC values in cereals were found to be lower than in clearings, rape, alfalfa and set‐aside, both for the whole year and for the breeding season only (Table 4 and Fig. 1(b)). The highest NC values were observed in alfalfa. Furthermore, adults inhabiting alfalfa and cereals were consistently longer than those inhabiting clearings and set‐aside (Table 4 and Fig. 1(a)). Individuals inhabiting cereals had higher BCI values than those inhabiting set‐asides and clearings (Tables 1 and 4 and Fig. 1(c)).

**Table 2:** Parameters of the General Linear Modelling models used to analyse body length (BL), body condition index (BCI) and nitrogen content (NC) throughout the year and during the breeding season, including the proportion of variance explained by the model (*R* ^2^) and the regression coefficients (*β*, on a log scale) of the quantitative predictors

	Whole year			Breeding season		
	BL	BCI	NC	BL	BCI	NC
*R* ^2^	0.159	0.177	0.262	0.151	0.158	0.270
*β* Date	0.0005	0.0008	0.0001	0.0002	0.0005	−0.0000
*β* Relative abundance	−0.0002	0.0165	−0.0027	0.0007	0.0138	−0.0028
*β* NC	−0.0001	0.0002	NA	−0.0001	0.0002	NA

NA, not analysed.

**Table 3:** Effect of variables on body length, body condition index (BCI, males only) and stomach nitrogen content of adult voles

	Body length		BCI		Nitrogen content	
	χ^2^	*P*	χ^2^	*P*	χ^2^	*P*
All adult individuals					
Date	**10.968**	**0.001**	0.872	0.350	**9.641**	**0.002**
Year	**13.351**	**0.004**	0.969	0.809	2.361	0.501
Season r/n	**4.169**	**0.041**	**4.773**	**0.023**	0.451	0.502
Habitat	**16.464**	**0.005**	**14.980**	**0.010**	**65.530**	**0.000**
rA	3.097	0.078	3.822	0.051	**5.298**	**0.021**
Sex	**4.080**	**0.043**	NA	NA	0.0003	0.986
NC	1.506	0.220	1.380	0.240	NA	NA
Adults captured during breeding season only					
Date	**13.986**	**0.000**	2.557	0.110	**13.615**	**0.000**
Year	**13.356**	**0.004**	0.624	0.891	2.149	0.542
Habitat	**13.518**	**0.019**	**15.197**	**0.010**	**61.262**	**0.000**
rA	0.887	0.346	1.593	0.207	2.689	0.101
Sex	**4.465**	**0.035**	NA	NA	0.221	0.639
NC	1.649	0.199	0.905	0.341	NA	NA

GLM (normal distribution, log link function) analysis was used, with significant results at *P* < 0.05 shown in bold. Where a variable was not analysed, it is indicated by ‘NA’. rA, relative abundance (number of voles per 100 trap‐nights); season r/n, effect of reproductive/nonreproductive season.

**Table 4:** Analysis of differences in body length (BL), body condition index (BCI) and nitrogen content (NC) between habitats (Tukey post‐hoc test)

	Clearing	Forest†	Cereals	Rape†	Alfalfa	Set‐aside
Clearing	—		**NC**, BL, BCI		BL	
Forest†		—				
Cereals	NC, BL		—	NC	**NC**	**NC**, BL, BCI
Rape†			NC	—		
Alfalfa	BL		**NC**		—	NC, **BL**
Set‐aside			**NC**, BL, BCI		BL	—

Results for all individuals are shown to the right of the diagonal, whereas those for adults captured exclusively during the breeding season are to the left of the diagonal. Significant results at *P* < 0.05 are indicated as BL, BCI, NC; bold is used when *P* < 0.001. †BCI was not analysed in rape and forest.

**Figure 2:**
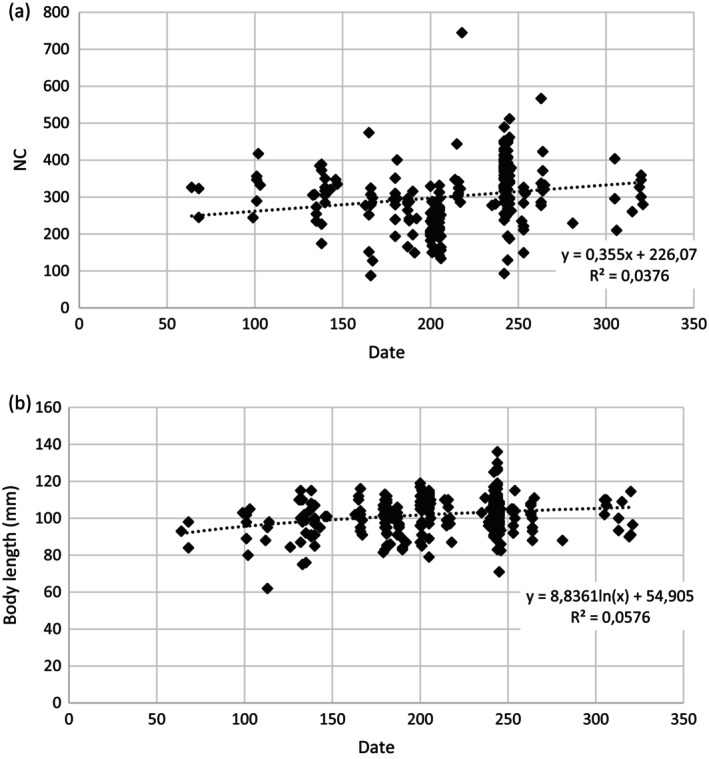
Relation between (a) stomach nitrogen content (NC, mg/g of dry body mass) and (b) body length (mm) of common voles and date of capture (days since the beginning of the year).

Year of capture had no effect on NC or BCI, but did affect body length (Table 3). Analysis of all years and breeding seasons shows that individuals were significantly shorter in 2008 than in other years (Table 5). A clear difference in body length and BCI was found between the breeding and non‐breeding seasons, because adults were longer and more robust during the breeding season (breeding season: mean body length = 102.1 mm, BCI = 0.280; non‐breeding season: body length = 98.8 mm, BCI = 0.239). Females were slightly shorter than males both throughout the year (mean length = 101.8 mm or females and 102.4 mm for males) and during the breeding season only (101.9 mm for females and 102.8 mm for males). Date of capture had a significant effect on NC and body length (Table 3). We tested for logarithmic effect, but the relationships are more complex. We can only conclude in a simplified way that NC increases during the season with body length peaking at intermediate dates (between May and July) throughout the year (Fig. 2).

**Table 5:** Analysis of differences in body length (BL) between years (Tukey post‐hoc test)

	2004	2005	2008	2010
2004	—		**BL**	
2005		—	**BL**	
2008	**BL**	**BL**	—	BL
2010			BL	—

Results for all individuals are shown to the right of the diagonal, whereas those for adults captured exclusively during the breeding season are to the left of the diagonal. Significant results at *P* < 0.05 are shown as BL, and bold type is used for *P* < 0.001.

**Figure 3:**
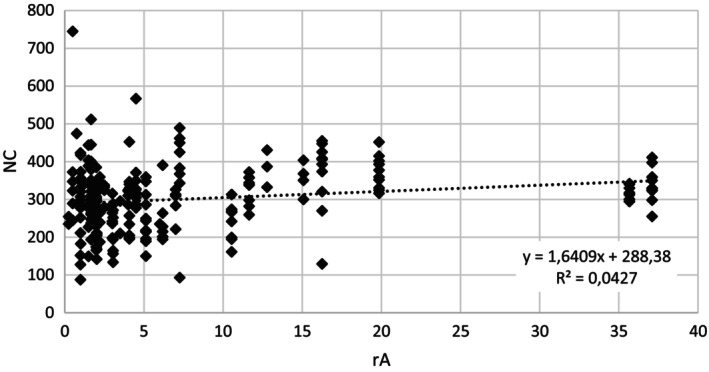
Dependence of body length (mm) on relative abundance (rA, number of captured common voles per 100 trap‐nights).

When the whole season was analysed, there was a positive relationship between the instantaneous rA in a given plot and individual NC (Table 3 and Fig. 3). However, when the analysis was restricted to the breeding season the relationship became non‐significant. Furthermore, the relationship between abundance and body size throughout the year cannot be clearly rejected, although the statistical significance is borderline. Interactions between the variables in the analysis of the whole year and the breeding season were tested; however, no significant effects were found.

## 4 DISCUSSION

It has been found that the quality of food and the body size of vole populations inhabiting different forests and agricultural areas differ significantly. However, the quality of food consumed does not appear to have a clear effect on the observed differences in body size in different habitats. Larger body sizes were observed in arable crops such as alfalfa, cereals and rape, in contrast to clearing, set‐aside and forest areas.

The foraging strategy of small mammals is strongly influenced by a combination of nutritional requirements and species mobility. Vole species are sedentary, use minimal space and do not exhibit substantial seasonal foraging movements.^44^, ^45^ The common vole is a typical herbivore, feeding on green plant biomass rather than seeds.^46^ The leaves of monocots and dicots are an important component of the vole diet, but the quantity and quality of green biomass and the diversity of the diet are strongly influenced by habitat and season, as evidenced by changes in food NC.^47^ Although NC in leaf biomass can vary between years,^48^ we did not detect an effect of the year of the study on the NC or BCI of the voles. We clearly confirmed that food quality differs between habitats. The highest NC in the vole diet was found in alfalfa, which has a high NC typical of legumes.^49^ The body length of voles in alfalfa was comparable with that of voles in rape and cereal fields. However, voles living in rape fields had a medium NC, whereas those living in cereal fields had the lowest NC of all habitats studied. Food quality was high in clearings and medium in forest and set‐aside, but voles were smaller in these habitats than in any agricultural crop. It can therefore be concluded that arable crops are the optimal environment for the body development of voles.

In general, reproduction, and consequently abundance, increase in periods and habitats with better food quality.^50^, ^51^, ^52^ Here we found a tendency towards a positive relation between rA and food quality, which was borderline also for body size. This relationship disappears when the analysis is restricted to the breeding season. Probably the main reason for this apparent discrepancy is lower food quality and smaller body size of individuals in the non‐breeding season, in the period of low abundance (in agreement with previous studies^53^, ^54^). These can strongly support the significance of the results when the non‐breeding season is included.

We did not find a clear relationship between abundance and body length. This relationship was only borderline significant in the analysis of the whole year. Larger body sizes at higher densities appear to be a general trend in small mammals with fluctuating abundance.^55^, ^56^ The analysed data set of voles contained a significant proportion of individuals from alfalfa, where high vole densities were observed. During the period of the highest densities, average rA for all alfalfa plots was 16.9 and the maximum recorded abundance was 37 individuals per 100 traps (in agreement with previous work^57^, ^58^). This is because alfalfa forms the most stable vegetation cover of all agricultural habitats and usually contains a significant proportion of weedy herbs that enrich the food spectrum.^59^ Alfalfa may also be preferred by voles because of its high NC, as protein may be a key factor influencing diet choice in small herbivores.^60^, ^61^ Although oilseed rape has medium NC values, the body length of voles was rather high. Like other overwintering and permanent crops (set‐aside, alfalfa, winter wheat), rape may play an important role in the overwintering of small mammals.^62^, ^63^, ^64^, ^65^ Rape is sown in autumn and provides high‐quality biomass during the winter. These good conditions also enable a good and early start of reproduction for voles.^65^ Cereals were also highly utilised by common voles and voles reached large body sizes despite the low NC of these crops. In cereals, most individuals were captured during the period from early June to early August, which corresponds to the stages of seed formation and ripening. During this period, the diet is high in starch and low in NC. The energy‐dense diet allows the individuals to grow considerably and accumulate sufficient body fat.

Set‐aside plots have been shown to act as refugia and trophic stores for animal pests during winter and other periods when the food supply from adjacent cropland is insufficient.^6^, ^66^, ^67^ However, it is likely that during the growing season, the cultivation of crops with fertilisers provides a more suitable food supply for increasing density and body development.^68^, ^69^, ^70^ By contrast, it has been found that forest environments are not optimal for common voles; they have low densities here, where they can survive and reproduce under certain conditions.^8^

As hypothesised, the body length of common vole populations showed interannual variability, with a decrease in size observed in 2008 compared with other years. In addition, low abundances were recorded in most habitats in 2008. The Chitty effect^12^ – a phenomenon that shows a correlation between larger body sizes at high densities – may provide an important explanation for this. Our populations have shown interannual oscillations in some habitats.^26^, ^27^, ^71^ In this study, a weak relation between the rA of the monitored population and body length was documented. An alternative explanation can be proposed, namely that in 2008, smaller and younger individuals than usually observed entered reproduction during the rising phase of the oscillation. It is hypothesised that they allocated more energy to reproduction than to body growth.^13^ Males were found to be significantly more likely than females to be oversized during the increase and peak periods.^24^ These large males were observed in 2004 and especially in 2005 when densities in the most used habitats, alfalfa and cereals, were extremely high. In 2008 our traps were dominated by females (37 females, 21 males) and no oversized individuals were caught. Differences between years may also be caused by climate or other factors affecting population demography. The harshness and length of winter, the amount and distribution of precipitation, temperature differences, vegetation conditions or, more generally, the quantity and quality of available food can affect reproduction and survival rates, and therefore overall population abundance.^17^, ^72^, ^73^

It appears that differences in body size between crops are not directly caused by differences in population density. The highest abundances of common voles were found in alfalfa and cereals, but also in set‐aside plots. The body size of individuals in alfalfa and cereals was large, but individuals in set‐aside are smaller than in agricultural habitats and their body size is comparable with voles from forests and clearings. Thus, we confirmed Jacob's results that the average body size of voles in arable landscapes was generally higher in more disturbed habitats (e.g. crop fields and pastures) than in less disturbed ones.^23^ The generally smaller body size of common voles in less intensively managed habitats may be caused by many factors, one of which may be predation pressure, in particular the abundance and species composition of predators. Predators survive better in less disturbed habitats, i.e. forests.^74^ Specifically, bird predators are more abundant in areas with trees and shrubs that provide nesting opportunities.^75^ The monitored habitats – clearings, forest and set‐aside – were always smaller plots in a diverse environment. It is known that ecotones with diverse vegetation cover generally have high abundance and activity of carnivores.^76^, ^77^ Furthermore, plots with perennial diverse vegetation provide good living conditions for weasels, which are the main terrestrial predators of voles.^78^, ^79^ This may result in higher predation pressure on voles on set‐aside, forest and clearings than on arable land. It has been confirmed that larger voles are subject to higher predation pressure.^78^ A negative relationship between mean body size and weasel abundance has even been observed.^78^, ^80^ In addition, small voles have an advantage over larger ones because they can pass through smaller holes than weasels.^78^

Other reasons for differences in body size between habitats may be food quality and availability.

The discrepancy between food NC and body size has several aspects. First, there are the limitations and simplicity of the method used (NIRS), which can only describe the actual quality of the diet. Even a perfectly balanced diet in an inadequate quantity will not ensure body growth. The NC of the diet is only one of the parameters describing the quality of the food, indicating the protein available. In small herbivores, NC can be a limiting factor in the rate of sexual and body growth of juveniles.^69^ However, even with adequate nitrogen saturation, organisms may lack some of its specific forms, particularly essential amino acids. Another limitation is that NC was measured only once, whereas the growth of the organism is a long‐term, continuous process. However, a lack of energy (fats, starch), micronutrients or vitamins can also have a significant negative impact on body size. Unfortunately, we cannot record all nutritional aspects of habitats, such as the range and abundance of weedy plant species, which are valuable sources of energy and micronutrients.^81^

Other habitat influences may also be relevant to vole demography. In addition to food supply and predation, habitats differ in other, often overlooked, environmental factors. These important factors may include the availability of shelter, the microclimate determined by the surrounding vegetation and location (shading, moisture, protection from temperature fluctuations), or human interventions related to habitat management (ploughing, mowing, application of chemicals directly affecting small mammals or vegetation cover, etc.). These habitat characteristics, whether natural or anthropogenic, influence both the morphological and physiological characteristics of individuals and the demography of the whole population.

Our results confirmed the hypothesis that the diet quality and body size of common vole populations vary significantly between different habitats. The largest individuals were found in intensively managed arable crops such as alfalfa and cereals, whereas smaller individuals were typical of set‐aside areas and clearings.

By contrast, the hypothesis that food quality relates to body size was not confirmed, because the largest body sizes were not consistently associated with the highest nitrogen values (e.g. in cereals individuals were large despite having the lowest food quality). This suggests that the body size of voles is influenced by more interacting factors than just food nitrogen alone, such as overall energy intake (e.g. starch‐rich diets), food quantity, habitat stability and management, predation pressure and microclimatic conditions.

Our findings ultimately support the hypothesis that body size and food quality are strongly influenced by the season. Although body length varied between years, the relationship between body length and abundance could not be clearly confirmed.

## 5 CONCLUSION

Body size and food quality were compared between vole populations living in forest and agricultural habitats. Our study demonstrates that body size can vary significantly between habitats, yet changes in body size are not primarily driven by the quality of the food consumed. The largest body sizes were observed in intensively managed arable crops such as alfalfa and cereals, which were not necessarily the habitats with the highest NC. By contrast, less intensively managed perennial habitats (set‐aside, clearings and forests) offered food richer in NC, yet voles here were smaller. This suggests that factors other than food quality play a more dominant role in shaping body size. From a pest management perspective, intensively managed crops appear to provide favourable conditions for vole development and may therefore increase pest potential. Larger body size can be associated with higher reproduction and survival rates, suggesting a higher risk of population increase and crop damage in these habitats.

## Data Availability

The data that support the findings of this study are available from the corresponding author upon reasonable request.
